# The role of the nurse in the care and management of patients with atopic dermatitis

**DOI:** 10.1186/s12912-020-00494-y

**Published:** 2020-11-04

**Authors:** Harmieke van Os-Medendorp, Elfie Deprez, Nele Maes, Sheila Ryan, Karina Jackson, Tonya Winders, Linda De Raeve, Christa De Cuyper, Steven Ersser

**Affiliations:** 1 Saxion, School of Health, Deventer, Enschede The Netherlands; 2 Department of Dermatology, University Hospital Ghent, Ghent, Belgium; 3 Department of Dermatology, University Hospital Limerick, Limerick, Ireland; 4 St John’s Institute of Dermatology, Guy’s and St Thomas’ Foundation NHS Trust, London, UK; 5 Allergy & Asthma Network / Global Allergy & Airways Patient Platform (GAAPP), Vienna, VA USA; 6 Department of Dermatology, Universitair Ziekenhuis Brussel (UZ Brussel), Brussels, Belgium; 7 Department of Dermatology, AZ Sint Jan, Brugge-Oostende, AV Belgium; 8 EADV-Nurse Association Working group Coordinator, Lugano, Switzerland; 9 Department of Nursing Science, Bournemouth University, Poole, UK

**Keywords:** Atopic dermatitis, Eczema, Treatment, Holistic care, Nurse specialist, Self-management, Education, E-health

## Abstract

**Background:**

The purpose of this paper is to provide an overview of key aspects of specialised dermatology nursing practice in the management of patients with moderate to severe atopic dermatitis. The role of dermatology nurse specialists in supporting patients and promoting disease understanding, education and treatment adherence continues to evolve. As features of specialised nursing care can also inform other nursing staff in a wide range of care settings, an overview of key components is examined. Observations presented are from a pan-European perspective and represent the collected view of a group of dermatology nurse specialists, dermatologists and patient advocates following two round-table discussions.

**Main body:**

Atopic dermatitis is a common, chronic, inflammatory disease characterised by erythematous/scaling skin lesions, with often intense pruritus. Disease course is cyclic with periodic disease flares of varying intensity, presenting management challenges to patients and families. Dermatology nurse specialists play a key role in providing education and substantial patient support to improve treatment outcomes and quality of life to patients and their family, delivered within a multidisciplinary team framework. Nurse-led education and 'eczema schools’ are of benefit in reducing disease severity and improving quality of life by enhancing self-management, adherence and patient engagement. eHealth tools, such as patient portals or online training platforms, can provide online learning, individualised education, and help to improve engagement. These and other initiatives, such as written action plans, are all essential to improve or maintain treatment adherence, self-management and quality of life.

**Conclusions:**

Dermatology nurse specialists play a central role in the assessment and management of moderate to severe atopic dermatitis patients and families. This places them in an ideal position to build strong and often long-term relationships with patients and parents. Such engagement promotes trust, assists in setting realistic expectations of treatment and outcomes, and enhances self-management and engagement in their own care. Providing emotional support, as well as formal and systematic education (including individualised practical advice) all contribute to improved treatment adherence and can enhance the quality of life of patients and their families throughout the course of this long-term condition.

## Background

The purpose of this article is to provide a contemporary overview of the key aspects of specialised dermatology nursing practice in patients with atopic dermatitis (AD) – also known as atopic eczema – and in particular to examine the evolving role of dermatology nurse specialists in supporting patients and promoting disease understanding, education and treatment adherence in those living with moderate to severe AD. A range of different nurse roles exist that may be involved in the care of AD patients, including general nurses working within dermatology, registered dermatology nurses, and nurses with additional training and responsibilities such as dermatology clinical nurse specialists, advanced nurse practitioners and nurse consultants. For simplicity in this manuscript we have used the term ‘dermatology nurse specialists’ to refer to those nurses with additional expertise in specialised dermatology nursing care. These observations are from a pan-European perspective formed from a group of specialist dermatology nurses (ED, NM, SR, KJ, SE), a dermatology nurse researcher (HvO), dermatologists (LDR, CDC) and a patient advocate (TW), who met to develop a consensus on selected topics of specialised dermatology nurse care in AD.

AD is a chronic, inflammatory skin disease characterised by erythematous and scaling lesions, often with intense pruritus [1]. Disease pathophysiology is complex, involving the interplay of genetic, environmental, and immunological factors. Defects in the epidermal barrier, often associated with gene mutations and polymorphisms in filaggrin and altered lipid metabolism, contribute to epidermal dehydration. This predisposes to immune responses to allergens, skin irritants and microbial proteins with subsequent acute and then chronic cutaneous inflammation. AD is common, affecting 20–30% of children, with most patients developing symptoms in infancy or early childhood. While most children will have spontaneous remission, persistence into adulthood is seen in approximately 30% of cases. The overall prevalence of AD in adults is between 2 and 8% and although adult-onset AD is well recognised, most have had symptoms since childhood [2–5]. In 80% of patients, an atopic background exists (“extrinsic/allergic AD”) with the remainder being non-allergic individuals (i.e., those with “intrinsic AD”) [6]. Patients with extrinsic AD are at higher risk of contracting other atopic diseases (allergic asthma, allergic rhino-conjunctivitis, and food allergy). Indeed, childhood onset AD is often a forerunner to the development of one or more atopic conditions in later life – the so called “atopic march” [7]. Other co-morbidities are also more common in AD patients. These include cardiovascular diseases, autoimmune diseases, low mood and issues with self-esteem [8, 9]. Psychological upset and sleep disturbance associated with skin irritation and pruritus have a substantial impact on quality of life (QoL) [10–13].

Typically, the disease course is cyclic with periodic exacerbations/disease flares of varying intensity [2]. The chronic relapsing disease pattern presents challenges for management, which involves ongoing treatment and monitoring for therapeutic response and tolerability. Treatment aims are to alleviate disease symptoms and reduce the frequency and severity of disease flares/exacerbations, to enhance QoL [14, 15]. A detailed summary of contemporary management approaches is beyond the scope of the present paper; for further detail on diagnosis and treatment, readers are encouraged to review key clinical guidelines and recommendations [3, 4, 14–17] and recent informative reviews [1, 2, 18]. Presented here are the most salient aspects which guide the role of specialised dermatology nursing care in the effective support of patients with AD.

## Main text

### Role of the nurse in the patient-centered care team

AD disease management may be delivered in different health settings (primary care, day-clinics, and secondary/tertiary care). The majority of patients have mild disease and are often managed in the primary care/community setting; only up to 10% are considered to have severe disease [14]. Specialist-led care is primarily delivered on an out-patient or day-clinic basis. Hospital admission for treatment is less common, although it may be provided for patients with severe or recalcitrant disease. In cases where a structured treatment and education program is considered necessary, in-patient care can help optimise topical therapy with out-patient follow-up, thereby reducing the need for systemic immunosuppressive or biologic therapy [16].

**Fig. 1:**
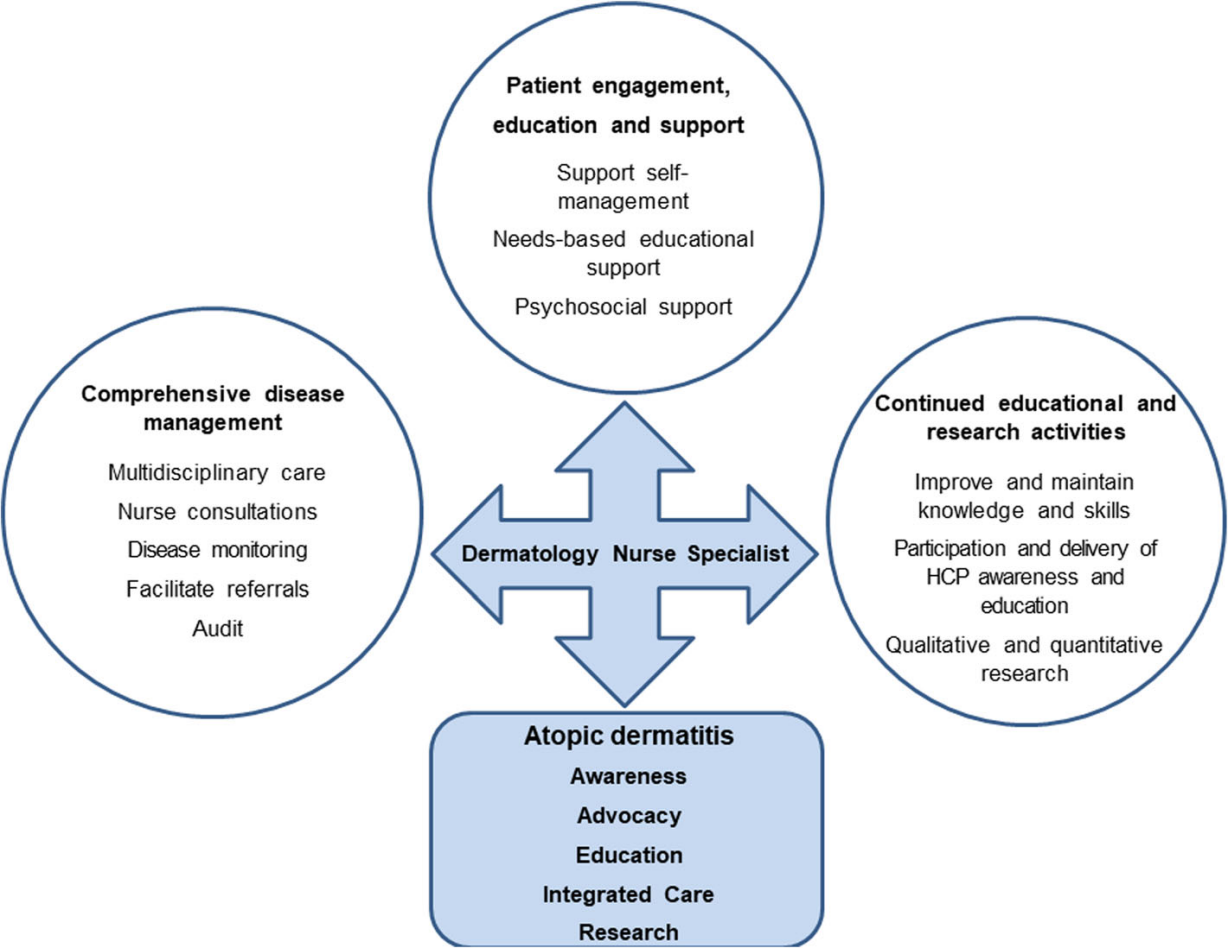
Overview of the role of dermatology nurse specialist in atopic dermatitis care

Nurses in all settings play a vital and valued role in providing care for the patient with AD [17, 18] (Fig. 1). Involvement of dermatology nurse specialists in providing and supporting clinical care allows the dermatologist to dedicate greater time to clinical aspects of patient consultations. The nurse –patient consultation addresses a range of aspects such as disease assessment, structured education and emotional support. However, no standardised format for consultations currently exists, nor consensus on what key elements should be included. On an individual patient basis, this may be informed by observations derived from multidisciplinary team discussions. A standardised nurse consultation ‘tool’ could be valuable as a framework to ensure that all appropriate relevant items are addressed and could allow a more consistent approach to monitoring disease course and patient support.

Ideally, moderate to severe AD care is delivered within a multidisciplinary team, with the focus on delivering person-centered care. In this integrated approach, communication, education, and treatment need to be individualised to suit the patient’s needs within a shared decision-making process. This must take into consideration personal disease history, severity and co-morbidities, and match realistic care expectations and personal preferences to the best scientific evidence available. Shared decision making can be supported by patient decision aids [19].

While the composition of the team varies between settings, institutions and across countries, dermatologists, dermatology nurse specialists, other nursing staff, pharmacists, along with psychologist or psychiatrist support, dieticians and other staff, all play a role [20]. Multidisciplinary initiatives and interventions, including education about the condition and its management, as well as psychological and behavioural programmes, may have a positive effect on outcomes in moderate to severe AD [21, 22].

Within such a team, nurses often serve as the principal contact between the patient (and their family) and the lead physician and other team members to support continuity of care [23]. Nurses tend to have well-established patient rapport and empathy skills, especially when addressing practical problems in eczema management, and are highly valued by patients [24]. They are, therefore ideally placed for continuity of primary patient contact at every stage of care provision, to both develop and document the patient’s clinical profile at the initial consultation and engage with patients through education and training throughout their care. In addition, dermatology nurse specialists may provide education and training to fellow healthcare workers, both within the team and in other settings, such as in primary care. Many are also actively involved in clinical research, integrated with clinical care.

Establishing and managing realistic patient expectations in their treatment and their role in self-management is fundamental. These may vary greatly in patients (and the parents of children) as may engagement in the shared decision-making process. Some parents/patients are highly motivated and informed, while others are more reluctant or cautious when seeking information and receiving appropriate clinical management; some may rely completely upon guidance from the healthcare team. Initial attitudes may change during the clinical disease course, and with patient experience and age. Providing patients/parents with the most appropriate and realistic information on their disease status, including the efficacy, safety, and suitability of proposed treatments, based upon their individual needs at each stage of the disease, is vital. Such information should be delivered in a clear, consistent fashion by all members of the healthcare team to inspire confidence and minimise confusion or misunderstanding on the part of the patient or parent [14, 15, 25–28].

Active engagement of the patient in decision making is paramount. A genuine sense of personal responsibility should be encouraged – especially regarding the importance of treatment adherence in achieving optimal self-management and optimal outcomes [19]. For example, while skincare and application of topical therapies is time consuming, and patient dissatisfaction contributes to poor treatment adherence, this may be overcome with support and education. Even with good understanding and acceptance of the chronic relapsing nature of moderate to severe AD, patients will naturally experience disappointment during adverse changes in their condition and nurses can offer reassurance and hope. Nurses can guide patients adapt through the disease cycle and therapy changes. Patients should be informed and understand that treatment escalation may be an effective and necessary strategy that is often only needed as a temporary measure to control disease exacerbations. Correct management of the medication, including frequency of administration and adaptation of the potency of the drugs/ointments, is crucial. Greater understanding will improve the patient’s adherence and therefore, enhance the clinical outcome.

### Overview of clinical management

Diagnosis of AD is based on family and personal history and clinical features. Most diagnostic criteria consider cutaneous aspects (including dry skin ± erythema, induration, and scaling), pruritus and a personal or family history of allergic skin or respiratory disease (i.e., AD, asthma or allergic rhino-conjunctivitis) as major diagnostic criteria, with disease chronicity and/or a relapsing pattern, and early onset (< 2 years of age) supportive of a diagnosis [29, 30]. Identification of additional trigger factors (food, environmental and job-related factors) is crucial in disease management. Establishing a complete profile of the patient is indispensable to determine the therapeutic strategy. Dermatology nurse specialists are often involved in skilled clinical assessment, gathering data to inform the diagnostic process and the estimation of disease severity and QoL impact.

Most treatment recommendations are based on disease severity and therapeutic response; a framework for categorizing AD severity as mild, moderate, or severe is important to understand and anticipate an individual’s treatment needs [14, 15, 25]. While treatment decisions can be complex, in part due to the wide variety of topical and systemic therapies available, most evidence-based clinical practice guidelines from specialist bodies in Europe and the US provide treatment recommendations on the basis of disease severity and treatment response [14, 15, 25–28].

Since AD is characterised by exacerbation and disease flares, followed by remission, severity should be assessed both at presentation and throughout the disease course; sequential measurements are ideal to generate reliable estimates [26]. A wide range of validated disease scoring tools are available, such as the Scoring Atopic Dermatitis Index (SCORAD) – with clinical and patient-oriented versions (PO-SCORAD), each assessing the extent and severity of skin involvement and also a subjective assessment of the severity of pruritus and sleep disturbance [31–33]. The Eczema Area and Severity Index (EASI) assesses the extent and severity of skin disease only [32]. Patient-reported outcome measures (PROMs) are also used for assessing symptoms or impact on QoL. These may be disease-specific – such as the validated Patient-Oriented Eczema Measure (POEM) and more generic dermatology-focused tools such as the Dermatology Life Quality Index (DLQI) [34–36]. Although these tools may not always be employed in the clinical practice setting, their use is on the increase and, by using them, nurses can contribute to the objective assessment to inform disease management and decisions about treatment and its evaluation. Discussing these measures with patients is often useful to provide context and help them gain a better understanding of their disease and its impact.

### Treatment aspects

The principles of treatment for moderate to severe AD are four-fold; (1) identification and avoidance of trigger factors; (2) skin rehydration to repair/restore the impaired skin barrier; (3) reducing itching/pruritus; (4) reducing skin inflammation, especially during disease flares, via topical and systemic therapies. Dermatology nurse specialists provide a crucial resource for patient education and support in all aspects of treatment.

Environmental irritants and known allergens can aggravate affected skin of AD patients and promote disease flares [14]. Well-recognised irritants include fabrics such as acrylic or wool, household chemicals (bleach or solvents), and fragrances. Daily use of emollients to reduce moisture loss is the mainstay of treatment. This has been shown to reduce pruritus and may also have a steroid-sparing effect, reducing the amount of topical corticosteroids (TCS) required [14, 37–39]. Specific skincare products such as emollients should be recommended, and according to personal needs lotions, creams or ointments that are preferred by the patient. Most over-the-counter products seem to be as effective as specialist prescription formulations [14]. Dermatology nurse specialists trained in AD management have a good knowledge of available products and are skilled in providing advice to patients who have difficulty in finding an emollient regime that suits their individual needs and preferences.

Key factors determining emollient use are the level of dryness of the skin and patient preference (i.e., one they are most likely to use). As individual preference is important to promote adherence [40]; it may be necessary to trial a range of products to establish a preferred combination of products. In principle, emollients should be applied directly on the skin after bathing and then applied on 2–3 further occasions throughout the day. Identifying the appropriate skincare regimen may take time, and once established, its use in everyday life may be time-consuming, and these aspects may be a source of patient or parental concern.

Wet-wrap therapy is a useful adjunct to standard skincare and topical therapies [41–44]. Their clinical value has been highlighted in Nicol’s robust large nurse-led study [41], although recent systematic review evidence calls for further research [42]. Wet wrapping involves the application of emollients or TCS, which are then covered by a layer of wet bandages or clothing and then by a further layer of dry clothing. Wet-wraps are especially useful in children as they provide a barrier to scratching affected skin in acute flares (although they are contraindicated when eczema is infected) [41–44]. A principal drawback is poor adherence, chiefly due to inadequate patient education. Nurse support in wet-wrap technique through demonstration is especially helpful, and particularly so for children, where engagement of both parent and child can be enhanced by incorporation of wet-wrapping into their lifestyle. Play strategies, such as “reward stickers” or wet-wrapping a favourite doll, can increase the acceptability of this technique to the child.

**Fig. 2:**
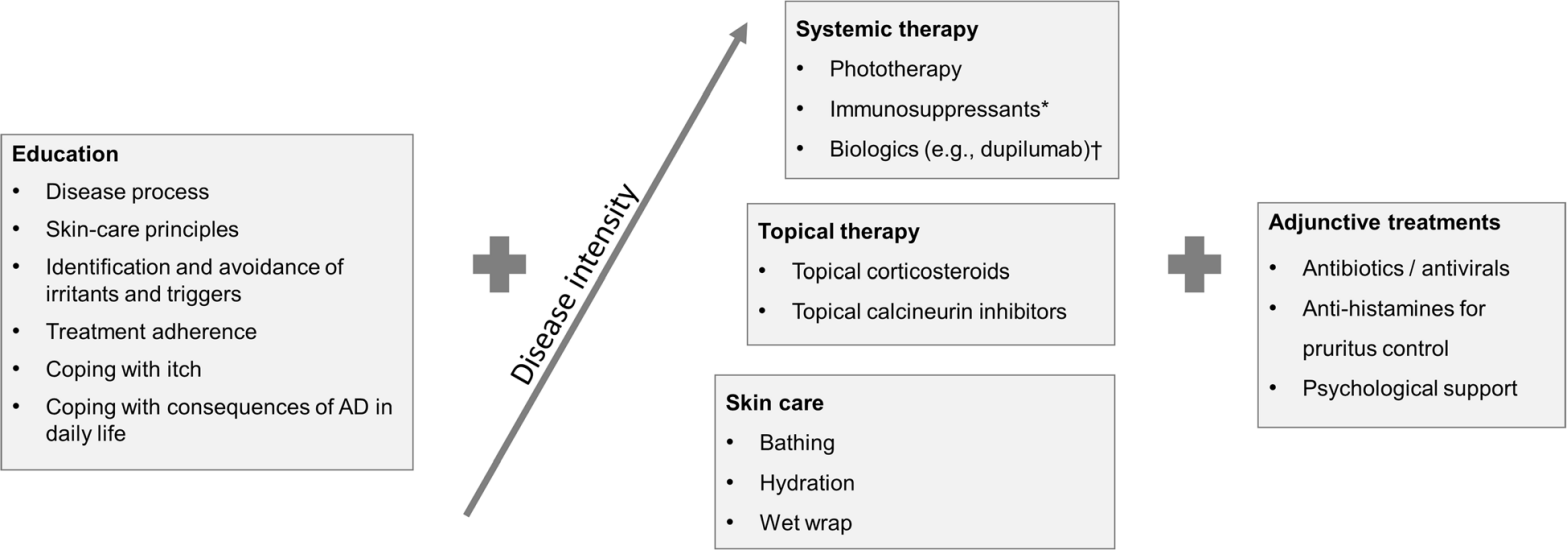
Overview of treatment principles in atopic dermatitis. Skin-care is essential and should be maintained regardless of any additional treatment. Topical corticosteroids (TCS) and topical calcineurin inhibitors (TCIs) are first-line therapies. A wide range of TCS preparations are available, and choice (potency, strength, dosage) should be tailored towards individual patient needs; typically, medium- to high-potency TCSs are used for short periods to treat acute flares. In sensitive areas (e.g. face, neck, flexural areas) lower-potency TCS or TCIs are recommended. *Systemic immunosuppressant therapy includes cyclosporine A (licensed therapy in Europe / off-label in the US) azathioprine, methotrexate, mycophenolate mofetil (all off-label) and systemic corticosteroids. †Biologic therapies e.g., dupilumab, now approved in Europe and the US in refractory patients with moderate-to-severe AD, either as monotherapy or in combination with TCS. Information based on published recommendations [3, 4, 14–17]

Medical therapies include topical agents, in particular TCS or topical calcineurin inhibitors (TCIs), phototherapy and systemic or biologic agents (immunosuppressants or biologic therapies e.g. dupilumab). Evidence-based guidelines recommend a stepwise approach to agent selection with a range of topical agents being considered as first-line before systemic immunosuppressants or biologic therapies are considered [14, 15, 25–28] (Fig. 2).

In general, the therapeutic approach is initial use of medium potency or higher-dose TCS preparations prior to TCIs. In severe AD, initial use of higher potency TCS may be preferred. When used appropriately TCS are safe; local side effects with longer-term use are uncommon and systemic side effects are rare. Nevertheless, safety may be an important aspect of patients often undue concerns or overt ‘steroid phobia’, especially in parents of affected children, which contributes to reduced treatment adherence. Explanation of the benefits and risks are, therefore, key aspects of patient education and reassurance [45, 46].

Dermatology nurse specialists are actively involved in delivering phototherapy to patients using established protocols, although treatment may be time-consuming for patients (and their families). Phototherapy is usually given in schedules of 3–5 weekly treatments for 6–12 weeks, with discontinuation if little response is seen within 2 months [14]. Treatment with existing care (emollients ± TCS) should be continued to minimise risk of acute flares. However, access to established centres may limit the utility of phototherapy to patients with moderate to severe AD who may benefit [14].

Patients with moderate-to severe AD may require systemic therapy with immunosuppressants (cyclosporine A, azathioprine, methotrexate, and mycophenolate mofetil) or targeted biologics such as dupilumab. Whilst decision-making should be shared between the patient and the physician to take account of patient preference, screening for the suitability of immunosuppressants or biologics, along with monitoring for adverse effects and clinical responses, are often coordinated by dermatology nurse specialists [15]. This may involve the ordering and review of various blood investigations according to local guidelines and protocols, and dermatology nurse specialists are increasingly involved in monitoring patients on therapies. When stepping-up treatment to systemic or biologic therapies, comprehensive discussions with patients or their family may be required to explain the rationale for such treatment and how duration of systemic treatment would be guided by clinical response and tolerability. Education on the more common side-effects of immunosuppressants or biologics and to provide perspective on the risk-benefit profile of specific agents is also important to raise awareness and reassure patients and their family. In addition, many of these agents may be given as subcutaneous injection (azathioprine, methotrexate, dupilumab) and while in some circumstances nurse-administration will be preferred, self-administration is a more practical option. As such, education and training in self-injection is essential.

### Promoting wellbeing

Patients with moderate to severe AD are at an increased risk of a wide range of co-morbidities, including cardiovascular, autoimmune, and psychiatric diseases [8, 9]. The systemic impact of AD is such that it has been considered by many – similar to psoriasis – to be a systemic disease [47]. An essential aspect of holistic, integrated care is the promotion of healthier lifestyles and supporting adherence with recommended medical care. This is important at all ages. For example, children with AD should be vaccinated in line with local or national vaccination schedules, although where possible, vaccines should be avoided during acute flares [14]. Healthy lifestyle changes may be required for some adult AD patients with moderate to severe disease [8, 9]. When appropriate, efforts to reduce alcohol use (and tobacco cessation) and promote weight-loss, with development of a regular exercise regime may all be important aspects of holistic patient care to improve wellbeing [9].

Other important aspects of wellbeing include the identification of those patients with a psychological disturbance. It should be recognised that adverse mood and poorer QoL can also develop in families of patients with moderate to severe AD. These may be formally measured and monitored by appropriate validated measures (e.g., Hospital Anxiety and Depression Scale) [48], although other factors beyond overt depression can be a particular burden. An important judgement is the need to assess when to instigate specialist referral to psychiatric or clinical psychology services.

Although beyond the scope of this article, a range of factors may adversely impact the patient’s sense of well-being. Sleep disturbance, self-esteem and social anxiety, intimate personal relationships and financial concerns due to treatment costs and impact upon work productivity and absences, can each contribute to lower mood and poorer disease-related life quality. Anxiety frequently relates to poor adherence to therapies, which may itself be associated with factors such as steroid aversion [49]. Nurses are well placed to provide counselling to patients and parents, actively listen; they may also use motivational interviewing to help minimise these impacts and support better adherence.

### Patient education

Nurses play a key role in the development and provision of systematic education on AD, both to patients, their relatives, and the wider healthcare community, on disease background, standards of care, and detailing risks and benefits of the available recommended treatments. In most therapeutic patient education (TPE) programmes, nurses often play a primary role in providing evidence-based and often individualised information to support self-management and promote active participation in shared decision making [20, 50–54]. Educational needs that are required to support self-care varies between patients, and needs-assessment can be difficult; simple scoring tools have been developed for this purpose [55]. While such education should be universal it is of particular importance in those patients with recalcitrant or refractory disease, or where treatment adherence or treatment concerns exist, and for those patients or families with poorer QoL, or who require additional psychological support [50, 52].

Two main service delivery models for education delivery exist; nurse-led focused group education and a broader multidisciplinary “eczema school” approach [50, 56]. To date, most experience and evaluation of the benefits of education is in children, where education may be directed towards children and their parents. There is no strong evidence that either approach is more effective as no direct comparative studies have been conducted. A recent Cochrane review of educational interventions in children concluded that either approach may lead to improvements in disease severity and QoL [56], although the eczema school approach is much more resource intensive. The “eczema school approach” was pioneered in Europe and has now also been used in the US with some success [57]. Eczema schools provide a structured intervention program for patients or parents of children with AD. Educational components include: disease information, practical advice on skin care and avoidance strategies, and nutritional advice. Psychological and social support are also provided. In the UK, nurse-led education such as the Eczema Education Programme (EEP) involving group education and social learning, has also been well received by parents and preliminary evidence indicates its clinical and QoL benefits [17, 58, 59], although further evaluative trial evidence is required. Although time-consuming, individualised education within an out-patient or day-clinic, may be more appropriate when delivering specific patient training on skincare and the use of topical therapies in adult patients with more complex educational needs.

Patients with moderate to severe AD may suffer a sense of isolation and learning about fellow patients’ experiences can help to alleviate this. The DIPEx (personal experiences of health and illness) international collaboration is a useful resource, providing videos of patient interviews on a wide range of medical conditions to provide genuine insight into the experiences of patients, family, and carers [60–62]. While patients should be encouraged to learn about their condition independently, − and the internet is a valuable source of information for some patients, − misleading or adverse impressions of appropriate therapy for moderate to severe AD can be made [63]. Nurses can provide guidance on appropriate reliable evidence-based information sources.

### eHealth strategies for management

Electronic digital health services (eHealth) – the use of information and communication technologies for health [64] – provide an important additional avenue to support AD care [65, 66]. In addition to assisting in scheduling appointments and prescription reminders, eHealth offers the ability to provide online medical consultations, with different studies reporting equivalent improvements in AD outcomes and QoL with online follow-up appointments compared with conventional face-to-face consultations [67–69].

eHealth strategies are considered favourably by both healthcare workers and patients, and nurses are an important contact in supporting access to eHealth resources. Such resources can range from static tools (providing core disease information) to more dynamic ones providing interactive online learning, self-management training or a personal ePortal providing patient-specific access to their electronic health records [70]. Mobile apps are available to monitor disease severity or symptoms such as itch [71, 72], and it is likely that the use of such tools by patients will increase.

Providing a suite of tools such as these can deliver individualised education and engagement [73, 74]. Increasingly, such tools are being developed and shared by national bodies or dedicated institutions e.g., those developed by the University Medical Center of Utrecht in the Netherlands or by the Foundation for Atopic Dermatitis [75, 76] In the US, the American College of Allergy, Asthma & Immunology, in partnership with the Allergy & Asthma Network patient organization, have developed an interactive tool to support patients with moderate-to-severe AD in shared decision making with their physician during a clinical consultation [77].

The educational and eHealth strategies described above can inform and support adherence. The chronic relapsing disease course, which requires rigorous attention to skincare, and different treatments over a prolonged period, can translate into a complex management plan, with episodes of disappointment and frustration regarding outcomes with topical therapies. This can result in reduced levels of adherence and subsequently poorer outcomes in moderate to severe AD.

The use of written eczema action plans, which provide patients or parents with a checklist of instructions, are also valuable to support self-management. Plans such as these are already an established aspect of asthma care, and although data on their effectiveness in AD is limited, they would seem to provide an important aspect to supporting care in AD and patient engagement [78–80]. Action plans for AD have been developed by a number of professional organizations, including the American Academy of Dermatology [81].

## Conclusions

Dermatology nurse specialists are key players in the assessment and management of patients with moderate to severe AD. Their central role within a multidisciplinary team places them in an ideal position to build strong and often long-term relationships with patients and parents. Such engagement promotes trust, assists in setting realistic expectations of treatment and outcomes, and enhances self-management and engagement in their own care. Providing emotional support, as well as formal and systematic education and individualised practical advice, may contributes to improved treatment adherence and can enhance QoL of patients and their families throughout the course of this chronic disease. The aspects described in this review may have value in educating non-specialist nursing staff in the evolving role of dermatology nurse specialists in moderate to severe AD patient management and holistic care.

## Data Availability

Not applicable.

## Abbreviations

AD: Atopic dermatitis
DLQI: Dermatology Life Quality Index
EASI: Eczema Area and Severity Index
EEP: Eczema Education Programme
PROMs: Patient-reported outcome measures
QoL: Quality of life
SCORAD: Scoring Atopic Dermatitis Index
TCIs: Topical calcineurin inhibitors
TCS: Topical corticosteroids
TPE: Therapeutic patient education
